# PDB_REDO: automated re-refinement of X-ray structure models in the PDB

**DOI:** 10.1107/S0021889809008784

**Published:** 2009-04-03

**Authors:** Robbie P. Joosten, Jean Salzemann, Vincent Bloch, Heinz Stockinger, Ann-Charlott Berglund, Christophe Blanchet, Erik Bongcam-Rudloff, Christophe Combet, Ana L. Da Costa, Gilbert Deleage, Matteo Diarena, Roberto Fabbretti, Géraldine Fettahi, Volker Flegel, Andreas Gisel, Vinod Kasam, Timo Kervinen, Eija Korpelainen, Kimmo Mattila, Marco Pagni, Matthieu Reichstadt, Vincent Breton, Ian J. Tickle, Gert Vriend

**Affiliations:** a Centre for Molecular and Biomolecular Informatics, NCMLS, Radboud University Medical Center, Nijmegen, The Netherlands; b CNRS/IN2P3, Laboratoire de Physique Corpusculaire, Université Blaize Pascal, Clermont-Ferrand, France; c Swiss Institute of Bioinformatics, Vital-IT Group, Lausanne, Switzerland; d Uppmax, SLV, Uppsala University, Sweden; e IBCP, CNRS Université de Lyon1, IFR128 BioSciences Lyon-Gerland, Lyon, France; f Institute for Biomedical Technologies Bari, CNR, Bari, Italy; g Fraunhofer-Institute for Algorithms and Scientific Computing, Sankt Augustin, Germany; h CSC – The Finnish IT Center for Science, Espoo, Finland; i Astex Therapeutics Ltd, Cambridge, UK

**Keywords:** X-ray crystallography, refinement, structure validation, Protein Data Bank, grid computing

## Abstract

Structural biology, homology modelling and rational drug design require accurate three-dimensional macromolecular coordinates. However, the coordinates in the Protein Data Bank (PDB) have not all been obtained using the latest experimental and computational methods. In this study a method is presented for automated re-refinement of existing structure models in the PDB. A large-scale benchmark with 16 807 PDB entries showed that they can be improved in terms of fit to the deposited experimental X-ray data as well as in terms of geometric quality. The re-refinement protocol uses TLS models to describe concerted atom movement. The resulting structure models are made available through the PDB_REDO databank (http://www.cmbi.ru.nl/pdb_redo/). Grid computing techniques were used to overcome the computational requirements of this endeavour.

## Abstract 2

The majority of previously deposited X-ray structures can be improved by applying current refinement methods.

## 1. Introduction   

The availability of three-dimensional macromolecular coordinates is a prerequisite for many types of studies, such as engineering protein function and stability, understanding the molecular origin of genetic disorders, and studying intermolecular interactions. For some research fields the accuracy of the coordinates is more important than for others. To understand whether a single nucleotide polymorphism causes an effect that leads to a disease, one often only needs to know its location in the protein, while the precise rotameric state of the amino acid side chain is of lesser importance. In structure-based drug design, on the other hand, even small inaccuracies in atomic coordinates can have detrimental effects on predictions of intermolecular contacts. Many methods in macromolecular structural bioinformatics are parameterized on the basis of known protein structures. For example, if the structures that were used to design a force-field are not very accurate, the force-field will not be very accurate either. Macromolecular crystallography methods have improved a lot in recent years (Kleywegt & Jones, 2002 ▶; Joosten *et al.*, 2007 ▶). The availability of rapidly increasing numbers of increasingly accurate protein structures is aiding the method development in fields such as drug docking (Nabuurs *et al.*, 2007 ▶), molecular dynamics (Hub *et al.*, 2007 ▶) and homology modelling (Krieger *et al.*, 2004 ▶).

Macromolecular structure models are stored and maintained by the worldwide Protein Data Bank (wwPDB; Berman *et al.*, 2003 ▶). The recent remediation of the existing PDB entries by the wwPDB has greatly improved the uniformity (Berman *et al.*, 2007 ▶). This has made it easier to extract data from the PDB in an automated fashion. Despite these efforts, the PDB suffers from problems of a fundamental nature (Hooft *et al.*, 1996 ▶). It is important that users of the PDB realize that they cannot blindly trust the entries. PDB entries are structure *models* that are the result of many iterations of trying to optimally explain indirect measurements. Structure validation tools such as *PROCHECK* (Laskowski *et al.*, 1993 ▶), *WHAT_CHECK* (Hooft *et al.*, 1996 ▶), *pdb-care* (Lütteke & von der Lieth, 2004 ▶) and the Electron Density Server (EDS; Kleywegt *et al.*, 2004 ▶) have shown that the PDB contains many anomalies, ranging from proteins with small deviations from normal geometry to structures that fit their submitted experimental data very poorly. A few obvious errors have led to structure retractions, *e.g.* the ABC transporters (Chang *et al.*, 2006 ▶), but the vast majority of structure anomalies remain in the databank. When anomalies in structure models are not recognized, this can have a serious impact on the quality of homology modelling and drug design.

It seems that often protein crystallographers lose their interest in a structure once it is deposited. This means that the person best suited to improve structure coordinates after an error is detected, or after better software has become available, is not very likely to actually work on the improvement. Therefore, an independent effort is required to try to apply new refinement techniques to the available structure models. This study shows that such an independent effort is possible. It is obvious that each PDB file has been refined with software that was at best state of the art at the moment it was published. Our previous studies in the field of NMR have shown that the re-refinement of existing PDB entries using methods that have improved over time can give significantly better structure models than the original ones (Nabuurs *et al.*, 2004 ▶). This project has led to an ongoing effort to re-refine all NMR structure models in the PDB for the RECOORD database (Nederveen *et al.*, 2005 ▶).

We describe here the re-refinement of all X-ray structure models in the PDB with resolution higher than 2.70 Å for which suitable experimental data are available. The goal of this massive re-refinement project is to develop a re-refinement protocol to obtain a better match between the experimental data and the atomic parameters (coordinates, displacement parameters) in the structure models. We use *R*-free (Brünger, 1992 ▶) as a measure of refinement progress and *WHAT_CHECK* as a tool to verify the quality of the coordinates. Because one single refinement protocol is used in this study, all the resulting structure models form a uniform data set in terms of refinement. A key feature of this protocol is the application of TLS models (Schomaker & Trueblood, 1968 ▶) that represent the translation, libration and screw displacement of groups of atoms that behave as (quasi) rigid bodies. Employing TLS models in the refinement process (Winn *et al.*, 2001 ▶) gives the benefit of anisotropic *B*-factor refinement without serious implications for the data/parameter ratio of the structure model. Care is taken to respect as much of the interpretation of the experimental data by the depositor as possible. In other words, alternate atoms are kept, ligands remain unaltered, residue types unmodified *etc*.

The re-refinement of such a vast number of structure models requires enormous amounts of computing power. Grid technology and large computer clusters are at present the best ways to get rapid access to vast numbers of CPUs for a relatively short period of time. Grid infrastructures are a collaborative environment sharing large numbers of often heterogeneous computing and storage resources distributed geographically. Their objective is to provide at any time readily accessible production quality resources. Because of the large number of CPUs available, they are ideally suitable for so-called ‘embarrassingly parallel’ applications, where computations can be easily split into fully independent tasks (Stockinger *et al.*, 2006 ▶). The re-refinement of 16 807 PDB files with 16 807 independent jobs each requiring from 1 to 24 h of CPU time is a very good example of an embarrassingly parallel project. The EMBRACE (European Model for Bioinformatics Research and Community Education, http://www.embracegrid.info) virtual organization of the EGEE infrastructure (Enabling Grids for E-sciencE, http://public.eu-egee.org) provides European bioinformaticians with computers, grid technology, and the support in terms of software and human expertise needed to perform massive computational projects such as the one we describe here.

## 2. Methods   

### 2.1. Data set selection   

All X-ray entries in the PDB for which experimental data of 2.70 Å resolution or higher were available before February 2007 were considered for re-refinement (Table 1 ▶). However, 4082 entries had incomplete experimental data files: the ‘_refln.status’ column was either missing or contained no useful information (all values were the same). Therefore the original *R*-free set for these entries could not be reproduced, which means that *R*-free cannot readily be used as an independent measure of model quality for these files. These incomplete entries were removed from the data set to avoid bias in the results. The resulting data set consisted of 16 807 PDB entries.

### 2.2. Re-refinement protocol   

The re-refinement procedure consists of three steps: data preparation, re-refinement and validation of the results. The procedure uses the *CCP4* suite (Collaborative Computational Project, Number 4, 1994 ▶): most notably *Refmac* (Winn *et al.*, 2001 ▶), *WHAT_CHECK* and a few dedicated programs. These dedicated programs as well as the re-refinement script described below are available for download at http://www.cmbi.ru.nl/pdb_redo.

#### 2.2.1. Data preparation   

We observed many inconsistencies in the reflection data files from the PDB that make automated use troublesome. For instance, there are several status flag schemes that separate *R*-free reflections from the working set (Table 2 ▶). In some cases, it was impossible to figure out the status flag scheme: for example, in 1au9 (Pantoliano *et al.*, 1989 ▶), which was originally refined without *R*-free. Data columns in reflection files do not always contain what is reported: for instance, the reflection data file for 1gq3 (Beernink *et al.*, 2009 ▶) reports phases in the ‘_refln.status’ column, and 1twi (Rajashankar *et al.*, 2002 ▶) has the reported intensities and amplitudes swapped. Estimated standard uncertainties for reflections are not always reported, or sometimes all the values are the same (*e.g.* 101m; Smith, 1999 ▶).

A program, *Cif2cif*, was written to reformat the reflection data to a consistent format, in which only the essential information was kept. The *Cif2cif* output contains reflection indices (*h*, *k*, *l*), amplitudes (*F*), estimated standard uncertainty values [σ(*F*)] and the *R*-free flag. When necessary, intensities and their σ values are converted to amplitudes. For consistency, the same method as described for the EDS (Kleywegt *et al.*, 2004 ▶) was used: *F* = *I* ^1/2^ and σ(*F*) = σ(*I*)/(2*F*). If σ(*F*) values are missing from the input file or when all values are zero, all σ values are set to 0.01 to avoid technical problems in *Refmac*.

A second program, *Extractor*, was written to combine relevant information from the experimental reflection data file and the coordinates file. These data included reported resolution and *R* factors, the resolution range of the experimental data, cell dimensions, and the TLS groups used in the original refinement. In cases where TLS was not used in refinement or where the TLS groups were not reported, they were defined as one single group per protein or nucleic acid chain.

The structure factor files were converted to MTZ format (a standard used in the *CCP4* suite) and used to recalculate *R* and *R*-free with *Refmac* using default settings. When needed, ligand topologies were automatically created by *Refmac*.

#### 2.2.2. Re-refinement   

Three types of re-refinement of increasing sophistication were used consecutively. Unless mentioned otherwise, default *Refmac* parameters as used in the *CCP4* graphical user interface *CCP4i* were applied. Two key exceptions were made: carbohydrate links were only used if they were described in the PDB file, and anisotropic displacement parameters were refined if ANISOU records were provided.

First, the structure models were subjected to ten cycles of rigid-body refinement. This was needed for a small number of structures that gave large deviations between the recalculated *R*-free and the value from the PDB header as a result of a rotation or translation of the coordinates with respect to the electron density: for instance, PDB entry 1akv (McCarthy *et al.*, 2002 ▶). As a side-effect of rigid-body refinement, *Refmac* removed all explicit H atoms.

The rigid-body refined structures were subjected to 20 cycles of restrained refinement, changing only the weight of the X-ray terms with respect to the geometric and displacement parameter restraints. Seven different weights were used: 1.00, 0.70, 0.50, 0.30, 0.10, 0.05 and 0.01. No noncrystallographic symmetry (NCS) restraints were used (see §3).

TLS refinement was performed on the rigid-body refined structures. Ten cycles of TLS refinement were performed, followed by 20 cycles of restrained refinement, changing only the weight of the X-ray terms.

The re-refinement resulted in 15 models for each PDB entry: one rigid-body refined structure model, seven structure models that were obtained through restrained refinement with TLS and seven models through restrained refinement without TLS.

#### 2.2.3. Selection and validation   

For both the set of re-refined structure models with TLS and the set without TLS, the best out of seven structure models was selected. The following criteria were used:

(1) All models with an r.m.s. deviation (rmsD) in the bond angles of over 3.0° from ideal were rejected.

(2) Models with a difference between *R* and *R*-free of over 0.05, *i.e.* 5 percentage points, were rejected. This rule was relaxed in cases where the difference between the recalculated *R* and *R*-free prior to re-refinement was also greater than 0.05. In those cases the requirement was that the difference was less than or equal to the initial difference.

(3) The model with the lowest *R*-free was selected from the remaining candidates. In the few cases with two or more models with the same *R*-free (up to three decimal places), the one with the smallest difference between *R* and *R*-free was selected.

(4) If the *R*-free value of the optimal model was higher than that of the rigid-body refined model, that is, when the structure model became worse as a result of re-refinement, all re-refined models were rejected and the rigid-body refined structure model was kept.

The best TLS-refined structure model was analysed with *WHAT_CHECK*. The structural *Z*-scores were compared with the values reported in the PDBREPORT databank for the original structure model. To ensure that all *Z*-scores were calculated with the same version of *WHAT_CHECK*, the entire PDBREPORT databank was recalculated for this project.

### 2.3. Grid implementation   

The re-refinements of the structure models were performed on a hybrid computing environment consisting of two virtual organizations (Biomed and EMBRACE) of the EGEE grid infrastructure and several clusters of EMBRACE-associated bioinformatics institutes in Europe. The infrastructure and especially the EMBRACE and biomed virtual organizations provided grid computing resources, while SIB (http://www.isb-sib.ch), IBCP (http://gbio-pbil.ibcp.fr), and UPPMAX/SNIC (http://www.uppmax.uu.se) provided additional computing resources on clusters. Each grid job consisted of 20 proteins that would run for approximately 20 h and were managed using the WISDOM production environment (Lee *et al.*, 2006 ▶; Jacq *et al.*, 2007 ▶). The maximum allowed runtime was 72 h for each job.

## 3. Results and discussion   

### 3.1. Re-refined structure models   

On a single CPU, the entire calculations would have taken about 17 years. With our grid and cluster computing approach more than 90% of the total calculation was finished in only two months – this shows the clear time advantage arising from the usage of modern computing technology. All 16 807 re-refinements were complete after four months; the vast majority were done after three weeks. The time delays were caused by problems at two of the grid nodes. As this was just as much a proof of concept for grid computing as for re-refinement, we decided not to use one of the available supercomputers to finish the whole job quickly.

After filtering for obvious outliers, 15 034 sets of re-refined structure models were obtained (Table 1 ▶). The majority of entries that could not be re-refined had problems with atom names in non-protein and non-nucleic acid compounds. Some of these compounds suffered from a lack of uniformity that made it impossible to use the existing topology files supplied with the *CCP4* package. Most of these problems have been solved by the remediation of the PDB (Henrick *et al.*, 2008 ▶), which was completed six months after the start of our project. The affected PDB entries will be redone in future re-refinements. Some other problems in the re-refinement were caused by size constraints in *Refmac* and time constraints on certain grid nodes.

### 3.2. Improved structure models   

#### 3.2.1. Change in *R* factors   

**Figure 1:**
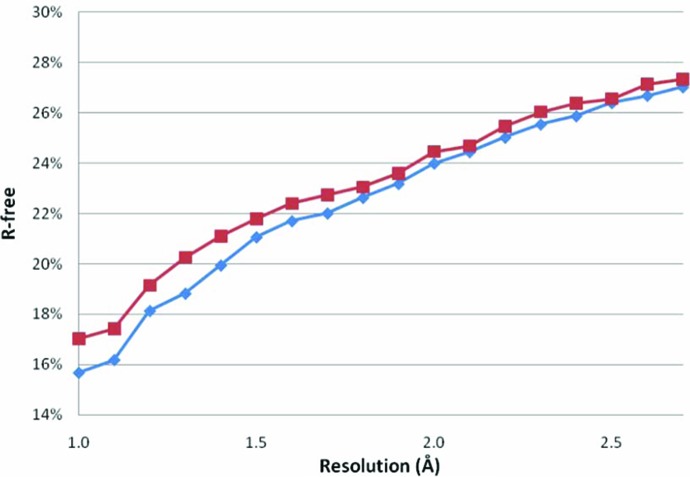
*R*-free values extracted from the PDB header (diamonds) and values recalculated with *Refmac* (Winn *et al.*, 2001 ▶) using the deposited experimental data (squares) plotted against the experimental data resolution. The values are averages for all structure models in 0.1 Å bins. The recalculated *R* values (not shown) follow the same pattern.

In the majority of cases, recalculation of *R*(-free) resulted in slightly higher values than the values extracted from the PDB header (Fig. 1 ▶). High-resolution structure models are affected more than low-resolution structure models. Many reasons for these deviations have been discussed previously by Kleywegt *et al.* (2004 ▶). Higher than expected values for *R*(-free) can, for example, occur because all experimental data were used in this study without resolution or signal-to-noise cut-offs, whereas the depositor may not have used all the high- and low-resolution reflections. Some extra complications were introduced by the *R*-free set. Deposition of experimental data with the wrong *R*-free set will cause a recalculated *R*-free value that is too low. The same happens when the *R*-free set was included in the final rounds of refinement before the structure model was deposited.

Subtle differences in the recalculated *R*(-free) value can also be caused by the conversion of reflection intensities to amplitudes. Several methods exist, but the method in the *CCP4* program *Truncate* (French & Wilson, 1978 ▶) is used most frequently. This method will be implemented in the re-refinement protocol.

Different solvent models may be used in refinement, resulting in different *R*(-free) values. Especially at high resolution, more sophisticated solvent models than the default model in *Refmac* may be used. Unfortunately, the applied solvent model cannot always be extracted from the PDB header. We are working on a method to circumvent this issue in the next refinement run.

The different ways in which the results of refinement with TLS models are deposited is more problematic. TLS tensors may be stored in the PDB header, but can also be added to individual (anisotropic) displacement parameters. Lack of uniformity in the PDB means that no single method can be used to recalculate *R*(-free) reliably when a deposited structure was refined with TLS models. For future calculations, we have adapted our re-refinement protocol to include several approaches for dealing with TLS and anisotropic displacement parameters. Because the TLS tensors and the displacement parameters are recalculated in the re-refinement, the final structure models are not affected by this issue.

The largest deviations in the recalculated *R*(-free) values are the result of rotations and translations of the structure model with respect to the electron density. Rigid-body refinement was used to correct this. In total 106 structure models with very large deviations (well over 10% in *R*-free) benefited from this approach; other structures had a small change in *R*(-free) or remained unaffected. Because only a small set of all evaluated PDB entries benefited from this rigid-body refinement, the re-refinement protocol was adapted to, in future calculations, only perform rigid-body refinement when *R*-(free) cannot be reproduced to within 5% from the value extracted from the PDB header.

**Figure 2:**
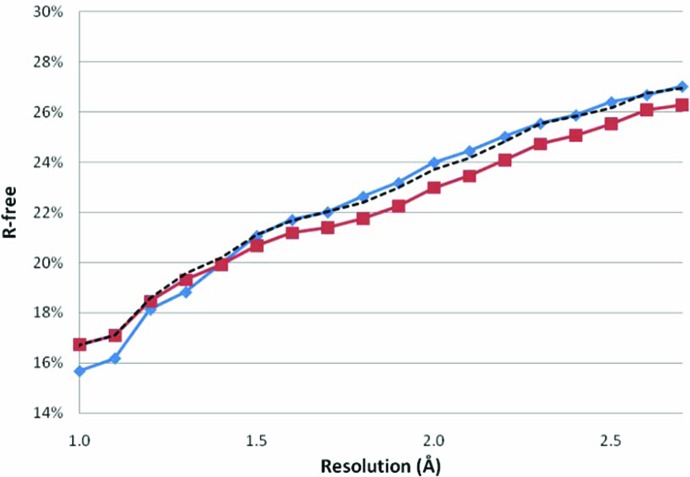
*R*-free values extracted from the PDB header (diamonds) and values obtained after re-refinement in *Refmac* (Winn *et al.*, 2001 ▶) with TLS models (squares) plotted against the experimental data resolution. The values are averages for all structure models in 0.1 Å bins. The effect of the TLS parameterization is clearly shown by the results of the re-refinement without TLS models (dotted line). For all but the highest resolution bins, refinement with TLS gives lower average *R*-free values.

Restrained refinement with TLS models gave a substantial improvement in terms of *R*-free (Fig. 2 ▶). A total of 10 046 structure models (67%) had a lower *R*-free than reported in the PDB header (Table 1 ▶). Restrained refinement without TLS had less effect: only 8012 structure models (53%) had a lower *R*-free than reported in the PDB header and the improvement was typically very small (Fig. 2 ▶).

#### 3.2.2. Structure quality validation   

The results in the previous section could lead to the conclusion that the *R*(-free) improvement is mostly the result of the TLS parameterization and no significant change of the atomic coordinates occurred. To assess this possibility, we performed a full structure validation with *WHAT_CHECK* of the original PDB entry and the optimal TLS-refined structure model to see the effect the re-refinement had on the coordinates. Validation is also needed to ensure that the observed improvements in *R*(-free) did not come at the cost of poorer geometry.

The *WHAT_CHECK* software provides a series of quality scores based on comparison with a set of about 500 PDB entries with a resolution of 1.4 Å or better. A comparison gives rise to a so-called *Z*-score. This score expresses the difference between the structure model and the test set as the number of standard deviations from the mean. A positive *Z*-score means that a structure model is better than the average of the test set. Among the different values that are calculated, the Ramachandran *Z*-score has often proven to be the best estimator of the geometric quality of a protein structure model (Laskowski *et al.*, 1993 ▶; Hooft *et al.*, 1997 ▶).

**Figure 3:**
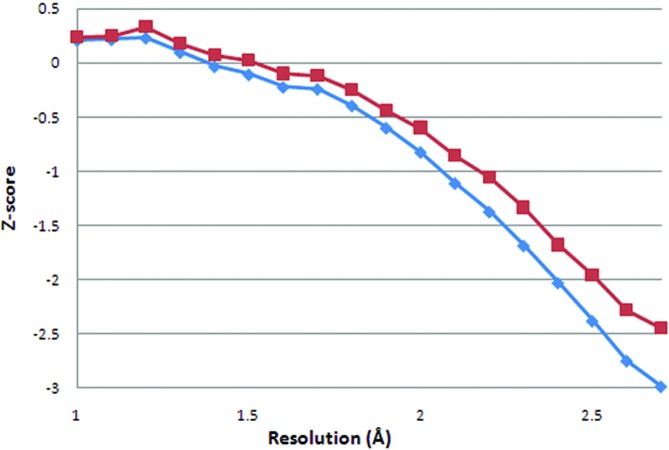
*WHAT_CHECK* Ramachandran plot appearance *Z*-scores (Hooft *et al.*, 1997 ▶) for original (diamonds) and TLS-refined structure models (squares) as a function of resolution. The values are averages for all structure models in 0.1 Å bins.

In Fig. 3 ▶, the Ramachandran *Z*-score before and after re-refinement is plotted against the resolution. There is a clear improvement of this quality score over the entire resolution range, which shows that the improvements in terms of *R*(-free) are backed by improved coordinates. *WHAT_CHECK* *Z*-scores for packing, side-chain rotamer normality and backbone normality were also evaluated but showed no significant improvement as a result of re-refinement. This can be expected since these scores are looking at atom arrangements in the medium-resolution range (1–5 Å); changes to the structure model of this magnitude are unlikely with our current automated re-refinement protocol. Typical atomic shifts are of the order of tenths of ångströms.

**Figure 4:**
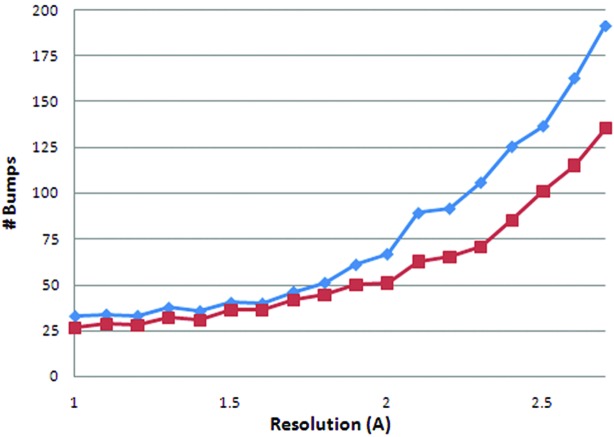
Atomic overlaps (bumps) per structure model as detected by *WHAT_CHECK* for original (diamonds) and TLS-refined structure models (squares) as a function of resolution. The values are averages for all structure models in 0.1 Å bins.

Another quality estimator is the average number of atomic overlaps or bumps. Like the Ramachandran *Z*-score, this estimator is sensitive to small changes in the atomic coordinates. Fig. 4 ▶ shows that re-refinement reduces the number of bumps for structure models over a wide resolution range.

The *WHAT_CHECK* validation reports showed many anomalies in the structure models both before and after re-refinement. Interpreting and, where possible, resolving these anomalies in an automated fashion will be the subject of further studies (Joosten *et al.*, 2009 ▶).

#### 3.2.3. The use of geometric restraints   

In X-ray refinement one of the important parameters is the relative weight of experimental X-ray terms and geometric restraints. Too much weight on the restraints results in structure models that sub-optimally describe the ‘real’ structure of a protein by hiding real bond-length (and -angle) deviations at important parts of the protein, *e.g.* the active site. Too little weight may result in structure models in distorted geometry. In effect, restraints should be kept as tight as necessary but as loose as possible (Kleywegt & Jones, 1995 ▶). This implies that the optimal restraint weight is resolution dependent: the higher the resolution, the weaker the restraints. The variation of the optimal restraint weights we found for each of the structures supports this, but the correlation between resolution and restraint weight is very weak. As a result, it is not possible to predict the optimal restraint weight.

**Figure 5:**
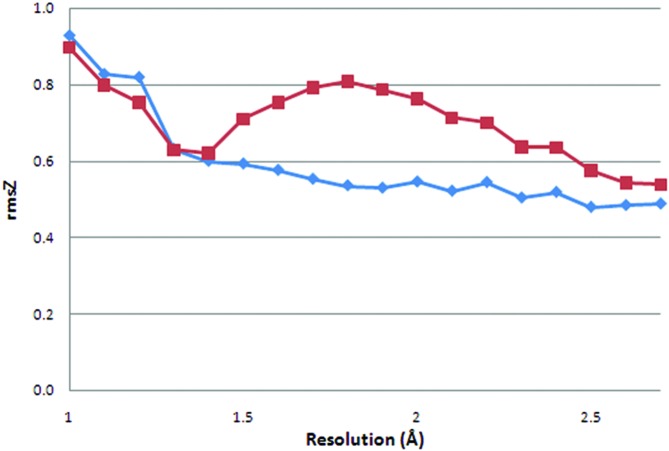
Bond-length r.m.s. *Z*-score per structure model as calculated by *WHAT_CHECK* for original (diamonds) and TLS-refined structure models (squares) as a function of resolution. The values are averages for all structure models in 0.1 Å bins.

Because our re-refinement protocol is aimed at lowering *R*(-free) without strict bond-length and bond-angle rmsD targets (the 3.0° cut-off on bond-angle rmsD is very liberal), the geometric deviations calculated from the re-refined structure models (Figs. 5 ▶ and 6 ▶) are less biased than the values extracted from the original PDB entries. Here, the r.m.s. *Z*-scores (rmsZ) are used as a measure of deviation from ideal instead of rmsD, because *Z*-scores reflect the different standard deviations for the ideal bond lengths and angles in the work of Engh & Huber (1991 ▶). For example, 0.01 Å deviations from ideal bond length between the Cα and Cβ atoms and the Cδ_1_ and C∊_1_ atoms of phenylalanine are treated the same with rmsD, whereas rmsZ acknowledges the difference between the two bond types. The latest version of *Refmac*, which was made available after we started our re-refinement, reports both rmsD and rmsZ. We have updated our protocol to use an rmsZ of 1.0 as a cut-off for the bond lengths and angles. An rmsZ value greater than unity means that the bond-angle deviation is larger than what can be expected based on the standard deviations in the restraint dictionary.

For re-refined structure models with a resolution of 1.7 Å or lower, there is clear resolution dependence for the average bond-length rmsZ: lower-resolution structures typically have a lower rmsZ (Fig. 5 ▶). The rmsZ value is expected to fall to zero at around 3 Å (about twice the average bond length, *i.e.* about 1.5 Å), because data of resolution *d* cannot contain information about interatomic distances less than *d*/2 (Tronrud, 2008 ▶). This is not observed here because only one important parameter for refinement, the relative weight of experimental X-ray terms and geometric restraints, was optimized in this study. Another key parameter for refinement, the relative weight of the displacement parameters and the X-ray terms, will be the subject of future experiments. The original PDB files do not show a clear decrease of rmsZ with decreasing resolution, but rather a constant value for structure models of 1.8 Å resolution or lower. This implies that a specific rmsD (or rmsZ) target was used with little attention to the X-ray resolution. The bond-angle rmsZ values follow the same pattern, but the difference between the re-refined and original structure models is much smaller.

At resolutions higher than 1.7 Å rmsZ goes up with increasing resolution for both bond lengths and angles in the original PDB entries. The re-refined structures do not follow this pattern. This is caused by the restraint settings used in our protocol: the highest setting (1.00) was still too low for some of the high-resolution structure models. This resulted in fewer structures than possible being improved in terms of *R*(-free) and in unexpected geometric rmsZ values. Our protocol was adapted to allow looser restraints at high resolution.

On average, atomic resolution structure models, 1.2 Å or higher (Sheldrick, 1990 ▶), in the PDB have bond-angle rmsZ values greater than 1.0. This is surprising because it means that the bond-angle deviations are larger than what is to be expected on the basis of the Engh and Huber parameters. This may be caused by the implementation of bond-angle restraints in *SHELX*(*L*) (Sheldrick & Schneider, 1997 ▶) which is commonly used at atomic resolution. The bond angles are restrained as one to three distances instead of actual angles. This approach is valid when the bond lengths are close to ideal, but this is not necessarily the case for refinement at high resolution. A different reason for these deviations to appear at atomic resolution should also be considered: there is some context-dependent variation in main-chain bond angles (Karplus, 1996 ▶). This variation may be underestimated by the Engh and Huber parameters because they are based on monomer and dimer small-molecule models that do not contain a macromolecular context. Only at atomic resolution is it possible to trust the experimental data enough to let such large deviations from the Engh and Huber parameters appear during refinement.

#### 3.2.4. Resolution dependence   

Fig. 2 ▶ shows that *R*-free for the TLS-refined structure models of 1.3 Å resolution or higher is higher than the value extracted from the PDB header. This is caused by the problems with reproducing the *R*-free value extracted from the PDB header (Fig. 1 ▶). When the recalculated *R*-free values are compared with values obtained for the TLS-refined structure models, it becomes clear that our re-refinement protocol works over the entire resolution range up to 2.7 Å. Notwithstanding, the method is indeed less successful at (near) atomic resolution than at lower resolutions. As discussed in the previous subsection, the restraint weights are probably the most important refinement parameter involved. The solvent model and the refinement of anisotropic dis­place­ment parameters may also improve the success rate of the refinement protocol.

Geometric quality in terms of Ramachandran *Z*-score or atomic overlaps (Figs. 3 ▶ and 4 ▶) shows greater improvements with decreasing resolution. This is not surprising because the lower-resolution structure models have more room for improvement. Real-space intervention is needed to improve these results.

PDB entries with resolutions lower than 2.7 Å were not considered in this study. At this resolution, NCS restraints become invaluable (Morris *et al.*, 2007 ▶). Unfortunately, NCS group definitions used in the original cannot be extracted from the PDB reliably. The relative weights of the NCS restraints are even harder to obtain. Redefining NCS groups and finding appropriate restraint weights based on the coordinates deposited in the PDB is not reliable: in the original refinement NCS may have been severely over- or under-restrained, which biases any NCS parameterization. The only alternative is a full NCS parameter optimization during the re-refinement. This was, at the start of our study, a very computationally intensive and lengthy process because it would involve a trial-and-error process. Fortunately, new automated methods have arisen that will greatly speed up the parameterization process (Smart *et al.*, 2008 ▶). Integration of this autoNCS method in the re-refinement protocol will allow for reliable re-refinement of structure models with NCS at resolutions lower than 2.7 Å. Preliminary tests with structure models without NCS show promising re-refinement results. Of course, at very low resolution our current method with refinement in Cartesian space becomes unsuitable and a method with torsion-space refinement must be implemented.

#### 3.2.5. Old PDB entries *versus* new   

**Figure 7:**
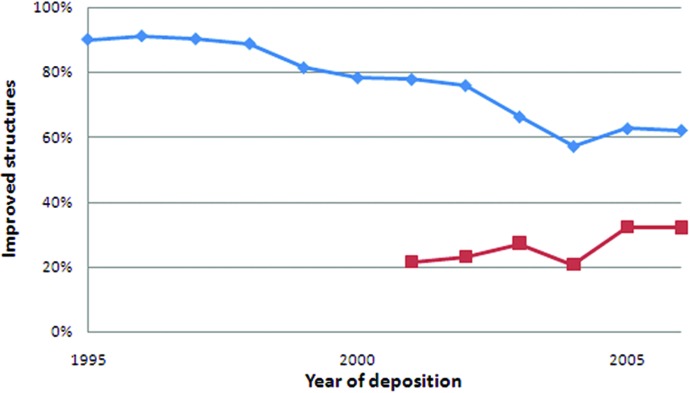
Percentage of structure models that improve in terms of *R*-free after TLS refinement plotted as a function of the year of deposition. The percentage of all evaluated structures (diamonds) decreases from 90% for 1995 to 62% for 2006. The percentage of structures previously refined with TLS (squares) varies between 21 and 32%.

Structure model quality, in terms of fit to the experimental data and in terms of geometry, has increased over time as new refinement methods arose (Kleywegt & Jones, 2002 ▶; Joosten *et al.*, 2007 ▶). It is to be expected that older structure models benefit more from re-refinement than newer structure models. The percentage of improved structure models plotted against the year of deposition supports this (Fig. 7 ▶). About 90% of the structure models deposited in 1995–1997 could be improved in terms of *R*-free. This percentage drops to just over 60% for structure models deposited in 2004–2006. These results show that the benefit of re-refinement is not limited to older structure models. Even 60% of recently deposited structure models can be improved upon re-refinement. It must be noted, however, that the average improvement in terms of *R*(-free) is smaller for recent structures than for structures deposited ten years ago.

The improvement of structure models previously refined with TLS shows no clear trend over the years (Fig. 7 ▶). TLS refinement may be too new to provide proper statistics at this point: for 2006, only 27% of all structure models were refined with TLS. The fraction of improved structure models varies between a fifth and a third. This success rate may be lower, but it remains clear that re-refinement is worthwhile for a large number of structure models. The success rate may be increased by re-evaluation of TLS group definitions.

## 4. Web site   

The re-refinement protocol and the re-refined structure models are available from http://www.cmbi.ru.nl/pdb_redo/. Each entry has a web page with a summary of the *R* factors after consecutive steps of re-refinement, a comparison between the *WHAT_CHECK Z*-scores before and after re-refinement, and information about the unit cell and structure factors. A compressed file with the structure models for each re-refinement step is provided. This file also contains an MTZ file for each structure model, which can be used to generate electron density maps. Links to the full *WHAT_CHECK* validation reports as well as links to relevant external databases, such as EDS and PDBsum (Laskowski *et al.*, 2005 ▶), are provided. Entries that are missing because our re-refinement procedure can (currently) not deal with the original PDB files are annotated in our new WHY_NOT server: http://www.cmbi.ru.nl/whynot/.

It should be noted that over the course of this project we encountered numerous problematic cases. One example is structure models for which the amplitudes in the submitted experimental data are inconsistent with the intensities in the same experimental data file. In such cases, both amplitudes and intensities have to be evaluated to see which set is correct. We are presently evaluating all files and validation reports to find ways to circumvent these problems, but the number of things that can go wrong is large, so this will be very time consuming.

## 5. Applications   

Re-refined structure models can, like the original ones, be used for drug design, molecular dynamics, structural biology and homology modelling. However, the structure model is not the only product of the re-refinement. An improved X-ray model gives rise to improved electron density maps. These can be used to (manually) inspect and solve problems recognized by validation software such as *WHAT_CHECK*. This can lead to further improvement of both geometric quality and fit to the experimental data. The results of this validation and real-space intervention effort are discussed elsewhere (Joosten *et al.*, 2009 ▶).

Re-refinement itself is a valuable means of testing refinement software. This does not only apply to *Refmac*; the development teams behind *Phenix* and *BUSTER-TNT* also regularly re-refine existing PDB entries to test their software and to understand the refinement problems that may occur (Adams, 2009 ▶; Joosten *et al.*, 2009 ▶).

## 6. Future work   

In this first complete re-refinement of all PDB files of 2.7 Å resolution or higher, the interpretation of the original depositors regarding amino acid sequence, alternate atoms, atomic occupancies, hetero compounds, water molecules *etc.* was left unaltered. We have started working on a re-refinement protocol without these constraints. Refitting of atoms to the electron density maps and error fixing based on *WHAT_CHECK* reports will be included. This will be an even larger effort that will require some new concepts, a lot of artificial intelligence and probably more than twice as much CPU time.

In our current work we have only evaluated PDB entries for which complete experimental data were available. Entries without a deposited *R*-free set were left out because any newly selected *R*-free set is not an independent measure of model quality. Eventually, these will be added too, using an adapted re-refinement procedure to (partially) compensate for the bias introduced by the newly selected *R*-free sets.

The TLS group assignments used in this work are very effective already but can be improved. Preliminary tests have shown that some structures that cannot be improved with our current TLS model can be improved using more sophisticated TLS group assignments like the ones from *TLSMD* (Painter & Merritt, 2006 ▶). At the moment, creating *TLSMD* groups is still computationally too expensive; we are working on a faster method.

There are, of course, many other issues to be resolved for fully automated re-refinement, both in the selection of the optimal result and in the parameterization of the refinement. The selection of the optimal re-refined structure model in this work was based on *R*-free. New versions of *Refmac* report the (log) free likelihood, which is a more appropriate target for optimization (Bricogne, 1997 ▶; Tickle, 2007 ▶). The refinement protocol has been updated to reflect this. The difference between *R* and *R*-free was used as a measure for over-refinement. The applied cut-off of 0.05 as maximum allowed difference is, although frequently used, rather arbitrary. A better method to check for over-refinement, which uses the ratio *R*-free/*R*, was described by Tickle *et al.* (1998 ▶). An adapted version of this method that uses *R*-free *Z*-scores has been added to the model selection step of our refinement method.

As mentioned before, optimizing the displacement parameter restraint weights in the re-refinement may lead to better results. The *Refmac* settings for these weights are by default not shown in the *CCP4* graphical user interface and good results can be obtained with the default settings. It is therefore likely that these settings are not always optimized. It has been shown that optimizing the *Refmac* displacement parameter restraints can lead to improved refinement results (Tickle, 2007 ▶).

In addition, refinement software continuously improves. For example, automatic NCS group optimization was recently made available, and the latest version of *Refmac* can deal with twinned crystal data sets without user interaction. These and other software improvements may not solve all problems, but they can make automated (re-)refinement methods a little better. We will continue to update our protocol to benefit from newly developed methods. The re-refinement protocol used here must therefore be seen as a starting point for further development, not as an attempt to build an alternative PDB.

## 7. Conclusion   

We have presented and thoroughly tested a re-refinement protocol for X-ray structure models that works over a wide resolution range. By employing methods such as TLS refinement, 10 046 out of 15 034 structure models (67%) are improved in terms of *R*-free. The geometric quality of the structure models, expressed as *WHAT_CHECK*’s Ramachandran *Z*-score, also increases. Both old and recently deposited PDB files can benefit from re-refinement.

These results show that re-refinement of existing PDB entries is worthwhile and, because the method is fully automated, little time investment is needed to re-refine a single structure model. We now routinely re-refine PDB entries before they are used for molecular dynamics, homology modelling or drug design.

## Figures and Tables

**Figure 6:**
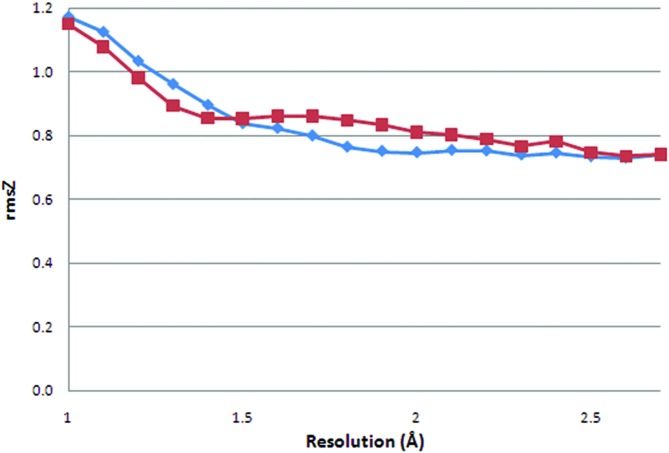
Bond-angle r.m.s. *Z*-score per structure model as calculated by *WHAT_CHECK* for original (diamonds) and TLS-refined structure models (squares) as a function of resolution. The values are averages for all structure models in 0.1 Å bins.

**Table 1:** Data set selection and re-refinement

PDB entries (January 2007)	41277
X-ray structure models	35003
X-ray + 2.70 resolution	29541
X-ray + experimental data (SF)	20889
X-ray + SF + *R*-free set	16877
X-ray + SF + usable *R*-free set	16807
Re-refined structure models	15034
Improved structure models	10046

**Table 2:** Status flag schemes for *R*-free set selection encountered in deposited reflection files at the wwPDB

Scheme	Working set	*R*-free set	Example PDB entry†
1	o	f	1aa6 (1dzi)
2	0	1	101m (1a4i)
3	1	1	1a8d
4	Positive integer	0	1b7d
5	Positive real number	0.00	1c3c
6	1.0	0.0	1a27

† PDB identifiers in parentheses are examples of reversed usage of the scheme.

## References

[bb1] Adams, P. (2009). Personal communication.

[bb2] Beernink, P., Endrizzi, J., Alber, T. & Schachman, H. (2009). In preparation.

[bb3] Berman, H. M., Henrick, K. & Nakamura, H. (2003). *Nat. Struct. Biol.* **10**, 980. 10.1038/nsb1203-980 14634627

[bb4] Berman, H. M., Henrick, K., Nakamura, H. & Markley, J. L. (2007). *Nucleic Acids Res.* **35**, 301–333. 10.1093/nar/gkl971 PMC1669775 17142228

[bb5] Bricogne, G. (1997). *Methods Enzymol.* **276**, 361–423. 10.1016/S0076-6879(97)76069-5 27799106

[bb6] Brünger, A. T. (1992). *Nature* (*London*), **355**, 472–475. 10.1038/355472a0 18481394

[bb7] Chang, G., Roth, C. B., Reyes, C. L., Pornillos, O., Chen, Y. J. & Chen, A. P. (2006). *Science*, **314**, 1875. 10.1126/science.314.5807.1875b 17185584

[bb8] Collaborative Computational Project, Number 4 (1994). *Acta Cryst.* D**50**, 760–763.

[bb9] Engh, R. A. & Huber, R. (1991). *Acta Cryst.* A**47**, 392–400.

[bb10] French, S. & Wilson, K. (1978). *Acta Cryst.* A**34**, 517–525.

[bb11] Henrick, K. *et al.* (2008). *Nucleic Acids Res.* **36**, D426–D433.

[bb12] Hooft, R. W. W., Sander, C. & Vriend, G. (1997). *CABIOS*, **13**, 425–430. 10.1093/bioinformatics/13.4.425 9283757

[bb13] Hooft, R. W. W., Vriend, G., Sander, C. & Abola, E. E. (1996). *Nature* (*London*), **381**, 272. 10.1038/381272a0 8692262

[bb14] Hub, J. S., Salditt, T., Rheinstädter, M. C. & de Groot, B. L. (2007). *Biophys. J.* **93**, 3156–3168. 10.1529/biophysj.107.104885 PMC2025676 17631531

[bb15] Jacq, N. *et al.* (2007). *Parallel Comput.* **33**, 289–301.

[bb17] Joosten, R. P., Chinea, G., Kleywegt, G. J. & Vriend, G. (2007). *Comprehensive Medical Chemistry II*, Vol. 3, edited by H. Kubinyi, pp. 507–530. Oxford: Elsevier.

[bb16] Joosten, R. P., Womack, T., Vriend, G. & Bricogne, G. (2009). *Acta Cryst.* D**65**, 176–185. 10.1107/S0907444908037591 PMC2631631 19171973

[bb18] Karplus, P. A. (1996). *Protein Sci.* **5**, 1406–1420. 10.1002/pro.5560050719 PMC2143451 8819173

[bb21] Kleywegt, G. J., Harris, M. R., Zou, J., Taylor, T. C., Wählby, A. & Jones, T. A. (2004). *Acta Cryst.* D**60**, 2240–2249. 10.1107/S0907444904013253 15572777

[bb19] Kleywegt, G. J. & Jones, T. A. (1995). *Structure*, **3**, 535–540. 10.1016/s0969-2126(01)00187-3 8590014

[bb20] Kleywegt, G. J. & Jones, T. A. (2002). *Structure*, **10**, 465–472. 10.1016/s0969-2126(02)00743-8 11937051

[bb22] Krieger, E., Darden, T., Nabuurs, S. B., Finkelstein, A. & Vriend, G. (2004). *Proteins*, **57**, 678–683. 10.1002/prot.20251 15390263

[bb23] Laskowski, R. A., Chistyakov, V. V. & Thornton, J. M. (2005). *Nucleic Acids Res.* **33**, 266–268. 10.1093/nar/gki001 PMC539955 15608193

[bb24] Laskowski, R. A., MacArthur, M. W., Moss, D. S. & Thornton, J. M. (1993). *J. Appl. Cryst.* **26**, 283–291.

[bb25] Lee, H.-C., Salzemann, J., Jacq, N., Chen, H.-Y., Ho, L.-Y., Merelli, I., Milanesi, L., Breton, V., Lin, S. C. & Wu, Y.-T. (2006). *IEEE Trans. Nanobiosci.* **6**, 288–295. 10.1109/tnb.2006.887943 17181029

[bb26] Lütteke, T. & von der Lieth, C. W. (2004). *BMC Bioinformatics*, **5**, 69. 10.1186/1471-2105-5-69 PMC441419 15180909

[bb27] McCarthy, A. A., Walsh, M. A., Verma, C. S., O’Connell, D. P., Reinhold, M., Yalloway, G. N., D’Arcy, D., Higgins, T. M., Voordouw, G. & Mayhew, S. G. (2002). *Biochemistry*, **41**, 10950–10962. 10.1021/bi020225h 12206666

[bb28] Morris, R. J., Perrakis, A. & Lamzin, V. S. (2007). *Macromolecular Crystallography – Conventional and High-throughput Methods*, edited by M. R. Sanderson & J. V. Skelly, pp. 155–172. Oxford University Press.

[bb29] Nabuurs, S. B., Nederveen, A. J., Vranken, W., Doreleijers, J. F., Bonvin, A. M., Vuister, G. W., Vriend, G. & Spronk, C. A. (2004). *Proteins*, **55**, 483–486. 10.1002/prot.20118 15103611

[bb30] Nabuurs, S. B., Wagener, M. & de Vlieg, J. (2007). *J. Med. Chem.* **50**, 6507–6518. 10.1021/jm070593p 18031000

[bb31] Nederveen, A. J., Doreleijers, J. F., Vranken, W., Miller, Z., Spronk, C. A. E. M., Nabuurs, S. B., Güntert, P., Livny, M., Markley, J. L., Nilges, M., Ulrich, E. L., Kaptein, R. & Bonvin, A. M. J. J. (2005). *Proteins*, **59**, 662–672. 10.1002/prot.20408 15822098

[bb32] Painter, J. & Merritt, E. A. (2006). *Acta Cryst.* D**62**, 439–450. 10.1107/S0907444906005270 16552146

[bb33] Pantoliano, M. W., Whitlow, M., Wood, J. F., Dodd, S. W., Hardman, K. D., Rollence, M. L. & Bryan, P. N. (1989). *Biochemistry*, **28**, 7205–7213. 10.1021/bi00444a012 2684274

[bb34] Rajashankar, K. R., Ray, S. S., Bonanno, J. B., Pinho, M. G., He, G., De Lencastre, H., Tomasz, A. & Burley, S. K. (2002). *Structure*, **10**, 1499–1508. 10.1016/s0969-2126(02)00880-8 12429091

[bb35] Schomaker, V. & Trueblood, K. N. (1968). *Acta Cryst.* B**24**, 63–76.

[bb36] Sheldrick, G. M. (1990). *Acta Cryst.* A**46**, 467–473.

[bb37] Sheldrick, G. M. & Schneider, T. R. (1997). *Methods Enzymol.* **277**, 319–343. 18488315

[bb38] Smart, O. S., Brandl, M., Flensburg, C., Keller, P., Paciorek, W., Vonrhein, C., Womack, T. O. & Bricogne, G. (2008). Abstracts of the Annual Meeting of the American Crystallographic Association, Knoxville, Tennessee, USA.

[bb39] Smith, R. D. (1999). PhD thesis, Rice University, USA.

[bb40] Stockinger, H., Pagni, M., Cerutti, L. & Falquet, L. (2006). *2nd IEEE International Conference on e-Science and Grid Computing* (*e-Science 2006*), pp. 58–65. Amsterdam: IEEE Computer Society Press.

[bb41] Tickle, I. J. (2007). *Acta Cryst.* D**63**, 1274–1281. 10.1107/S0907444907050196 18084075

[bb42] Tickle, I. J., Laskowski, R. A. & Moss, D. S. (1998). *Acta Cryst.* D**54**, 547–557. 10.1107/s0907444997013875 9761849

[bb43] Tronrud, D. E. (2008). Abstracts of the Annual Meeting of the American Crystallographic Association, Knoxville, Tennessee, USA, and personal communication.

[bb44] Winn, M. D., Isupov, M. N. & Murshudov, G. N. (2001). *Acta Cryst.* D**57**, 122–133. 10.1107/s0907444900014736 11134934

